# Patient-derived cell lines and orthotopic mouse model of peritoneal carcinomatosis recapitulate molecular and phenotypic features of human gastric adenocarcinoma

**DOI:** 10.1186/s13046-021-02003-8

**Published:** 2021-06-23

**Authors:** Shumei Song, Yan Xu, Longfei Huo, Shuangtao Zhao, Ruiping Wang, Yuan Li, Ailing W. Scott, Melissa Pool Pizzi, Ying Wang, Yibo Fan, Kazuto Harada, Jiankang Jin, Lang Ma, Xiaodan Yao, Namita D. Shanbhag, Qiong Gan, Sinchita Roy-Chowdhuri, Brian D. Badgwell, Zhenning Wang, Linghua Wang, Jaffer A. Ajani

**Affiliations:** 1 Department of Gastrointestinal Medical Oncology, The University of Texas MD Anderson Cancer Center, 1515 Holcombe Blvd, Houston, TX 77030 USA; 2 Department of Surgical Oncology and General Surgery, First Hospital of China Medical University, Shenyang, 110001 P. R. China; 3 Department of Genomic Medicine, The University of Texas MD Anderson Cancer Center, Houston, TX 77030 USA; 4 Department of Pathology, The University of Texas MD Anderson Cancer Center, Houston, TX 77030 USA; 5 Department of Surgical Oncology, The University of Texas MD Anderson Cancer Center, Houston, TX 77030 USA

**Keywords:** Gastric adenocarcinoma, Peritoneal metastases, Patient-derived cell lines, Patient-derived xenograft, molecular profile, Patient-derived orthotopic model

## Abstract

**Background:**

Gastric adenocarcinoma with peritoneal carcinomatosis (PC) is therapy resistant and leads to poor survival. To study PC in depth, there is an urgent need to develop representative PC-derived cell lines and metastatic models to study molecular mechanisms of PC and for preclinical screening of new therapies.

**Methods:**

PC cell lines were developed from patient-derived PC cells. The tumorigenicity and metastatic potential were investigated by subcutaneously (PDXs) and orthotopically. Karyotyping, whole-exome sequencing, RNA-sequencing, and functional studies were performed to molecularly define the cell lines and compare genomic and phenotypic features of PDX and donor PC cells.

**Results:**

We established three PC cell lines (GA0518, GA0804, and GA0825) and characterized them in vitro. The doubling times were 22, 39, and 37 h for GA0518, GA0804, and GA0825, respectively. Expression of cancer stem cell markers (CD44, ALDH1, CD133 and YAP1) and activation of oncogenes varied among the cell lines. All three PC cell lines formed PDXs. Interestingly, all three PC cell lines formed tumors in the patient derived orthotopic (PDO) model and GA0518 cell line consistently produced PC in mice. Moreover, PDXs recapitulated transcriptomic and phenotypic features of the donor PC cells. Finally, these cell lines were suitable for preclinical testing of chemotherapy and target agents in vitro and in vivo*.*

**Conclusion:**

We successfully established three patient-derived PC cell lines and an improved PDO model with high incidence of PC associated with malignant ascites. Thus, these cell lines and metastatic PDO model represent excellent resources for exploring metastatic mechanisms of PC in depth and for target drug screening and validation by interrogating GAC for translational studies.

**Supplementary Information:**

The online version contains supplementary material available at 10.1186/s13046-021-02003-8.

## Introduction

Gastric adenocarcinoma (GAC) is the fifth most common cancer and the third leading cause of cancer-related mortality worldwide [1]. GAC is usually diagnosed in late stages, treatments have limited effectiveness, and innate therapy resistance contributes to high mortality [2, 3]. Peritoneal carcinomatosis (PC) is frequently identified in advanced GAC, and the 5-year survival rate of GAC with PC is < 5% [4]. Despite some progress in systemic therapy, the overall survival of GAC patients with PC has not improved appreciably [3, 5–8]. Once a patient develops PC, their clinical course is often downhill with many mounting symptoms [8, 9]. PC is inherently therapy resistant and patients often succumb to PC within 6 months. We have recently reported on profiling of PC cells [10] and the role of YAP1 in PC progression [11]. However, much more in depth analysis is needed to discover novel targets/drugs for treating patients with PC.

The resources to study PC are starkly limited. Patient-derived tumor cell lines, patient-derived xenografts(PDXs), and orthotopic models will be indispensable in this respect. There are 38 gastric cancer cell lines in the cancer cell line encyclopedia (CCLE); the majority of these are from Asian patients [12]. However, studying GAC remains a challenge because it is also a heterogeneous disease [13, 14], and the current cell line bank may not reflect the phenotypic diversity and cellular heterogenicity of GAC in the western countries. Therefore, additional resources would benefit the research community.

Several human GAC cell lines have been used to create orthotopic mouse models of PC [15–20]. However, most of these cell lines (e.g., NCI-N87, MKN-45, MKN-28, AGS, Snu-16) or patient-derived cells (e.g., GC04, GC07, GC10, GTX-085) have not formed PC after orthotopic implantation unless manipulated [15–19]. Yanagihara K et al. established several subclones from two PC cell lines by repeated selection (10-cycles) and formed PC only 30% of the time and took several months before PC could be identified [20]. Thus, a PC model with high efficiency and a representative phenotype that can closely mimic the clinical course of advanced GAC would be an advantage for translational studies.

Here, we report on three new GAC cell lines established from PC cells from the Western patients. All three patient-derived PC cell lines are highly tumorigenic in the subcutaneous PDX model. Furthermore, whole exome sequencing (WES) and RNA sequencing (RNA-seq) revealed that the PDXs recapitulate the transcriptomic profile and conserve some of the mutational and copy-number variations (CNVs) similar to donor PC cells. Moreover, these cell lines are suitable for studying cytotoxics and target inhibitors. Most importantly, one cell line (GA0518) reliably formed PC in mice within 30 days in the PDO model. Thus, these new cell lines and the PDX and PDO models provide an opportunity to investigate mechanisms of PC and validate therapeutic targets.

## Materials and methods

### Establishment of novel GAC cell lines from PC cells and PDX

Three PC cells were collected from volunteering patients with GAC and malignant ascites; and patient clinical characteristics including therapy, tumor types, site of metastases, and histopathology are listed in Supplemental Table 1. Both GA0518 and GA0825 cell lines were established from PDXs that were generated by a subcutaneous injection of donor PC cells (IP-013 and IP-116) respectively. In detail, after red blood cell lysis and PBS wash, 5 million PC cells from patient IP-013 (for GA0518) and patient IP-116 (for GA0825) were subcutaneously injected into the back flanks of severe combined immunodeficiency (SCID) mice. Tumors from mice were dissected 4 weeks post injection, and disassociated cells from the tumors were directly cultured in RPMI-1640 medium with 7% FBS and 1% antibiotics. After more than 10 passages, the cells were considered as stable cell line. For GA0518 cells derived from IP-013 PC cells, the cell line was established from the first-generation PDX, and then the cells were reinjected into the SCID mice (second generation) and propagated further. In contrast to GA0518 and GA0825 cell lines, the GA0804 cell line could be established directly from donor PC cells (patient IP-107-02) via culture in RPMI medium (7% FBS, 1% antibiotics) and expanding them for more than 10 passages.

### Improved PDO mouse model

To assess orthotopic tumor growth and PC in mice, we first transduced the cell lines with a lentivirus expressing Luciferase and mCherry for in vivo bioluminescence (BLI) imaging of tumor growth in SCID mice. Luciferase-expressing cells (1 × 10^5^ cells in 30 μl in 50% Matrigel–phosphate-buffered saline) were injected into the distal stomach wall. We applied an improved orthotopic model to avoid the leakage into the peritoneal cavity (false positive). More details are provided in the results. The injection site and PC were monitored by in vivo BLI imaging weekly with intraperitoneal injection of 150 μg/g of D-Luciferin (PerkinElmer).

### Statistical analysis/statistics

Data were analyzed using the Student t-test and Fisher exact test (for colony formation and cell migration assays). Other assay results are presented as mean ± standard error of the mean and represent the results of at least three experiments. The significance of differences between groups was judged using a two-tailed Student t-test. Results were considered statistically significant if the *p* value was < 0.05. The statistical tests were done with GraphPad Prism 8 software (GraphPad Software).

More detailed materials and methods can be found in the online Supplementary Methods.

## Results

### Morphology and growth features of the cell lines

**Fig. 1:**
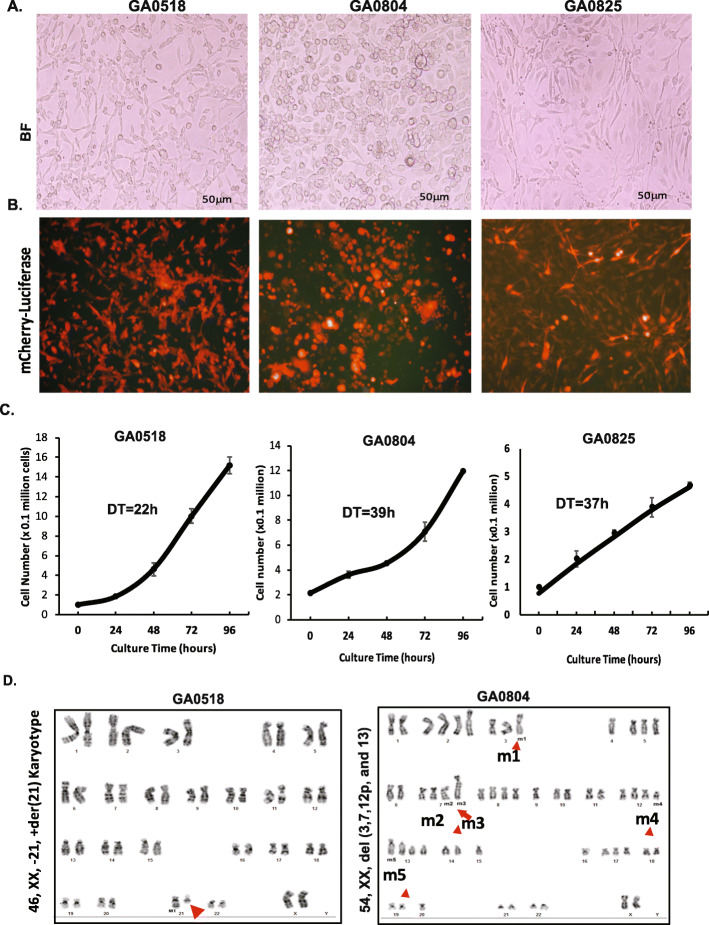
Characterization of the new GAC cell lines and in vitro growth features. **A** & **B**. Cell morphology of three new patient-derived GAC cell lines (GA0518, GA0804, and GA0825) was observed under a bright-field (BF) microscope (**A**), and cells stably transfected with mCherry-Luciferase were observed under immunofluorescence microscope (**B**). **C**. Doubling time for each of the three cell lines was estimated at the exponential phase according to Materials and Methods. **D**. Karyotyping analyses of GA0518 and GA0804 cells were performed by the Cytogenetics and Cell Authentication Core at MD Anderson as described in Materials and Methods. Abnormal chromosomal changes were observed in GA0518 (left) and GA0804 cells (right). Red arrows indicate some of the chromosomal changes

The three PC cell lines had different morphologies under the microscope (Fig. 1A and B, upper panel). In the 2D culture system, most GA0518 cells grew adherent as a monolayer of epithelial-like cells and spindle-like cells with about 5% of cells remained in suspension. GA0825 cells grew adherent as a monolayer of spindle shape and epithelial like with a few cells remained in suspension. While GA0804 cells grew and organized differently showing cobblestone like shape with ~ 10% grew in suspension (Fig. 1A). To visualize cell growth in vitro and in vivo, we stably transfected mCherry-Luciferase into each of the three cell lines to decipher the morphology more clearly and allow monitoring by BLI in vivo (Fig. 1B).

Doubling time was estimated during the exponential phase of growth. GA0518 cells had the shortest doubling time, 22 h. GA0804 and GA0825 cells had similar doubling times, around 39 h and 37 h, respectively (Fig. 1C).

### Karyotyping and molecular features of the cell lines and donor PC cells

GA0518 cell line showed a high level of heterogeneity with several chromosomal abnormalities that matched the donor sample (IP-013) with more mutations (Supplemental Fig. 1A). Their karyotype was heterogeneous and 70% cells had abnormal chromosomes including 40% with 45 chromosome and one X chromosome loss, designated as 45, X karyotype (Supplemental Fig. 1B, left); 20% with 46, XX with deletion in Chromosome 21q, designated as 46, XX,-21, +der [21] (Fig. 1D, left); and 10% with deletion in chromosome 8p and 21q, designated as 46, XX, del (8p), − 21, +der [21] (Supplemental Fig. 1B, right). In contrast, GA0804 line was more homogeneous but with 54 chromosomes and more structural rearrangements, including five structural chromosomal abnormalities and rearranged chromosomes, designated as 54, XX, del [3]; del [7]; del [7]; del (12p) and del [13] (Fig. 1D, right, red arrow). Finally, the karyotyping for commercial GAC cell line Snu1 showed an extra chromosome 20, a chromosomal rearrangement leading to long arms in one chromosome 1 and one chromosome 4 (Supplemental Fig. 1C). The designation of karyotyping for Snu-1 was: 47,XY, der(1)t(1;4)(q23;q33),der(4)del [4] which is similar to the Snu-1 karyotype previously described [21].

### Expression of CSC markers and oncogenic genes in cell lines and donor PC samples

**Fig. 2:**
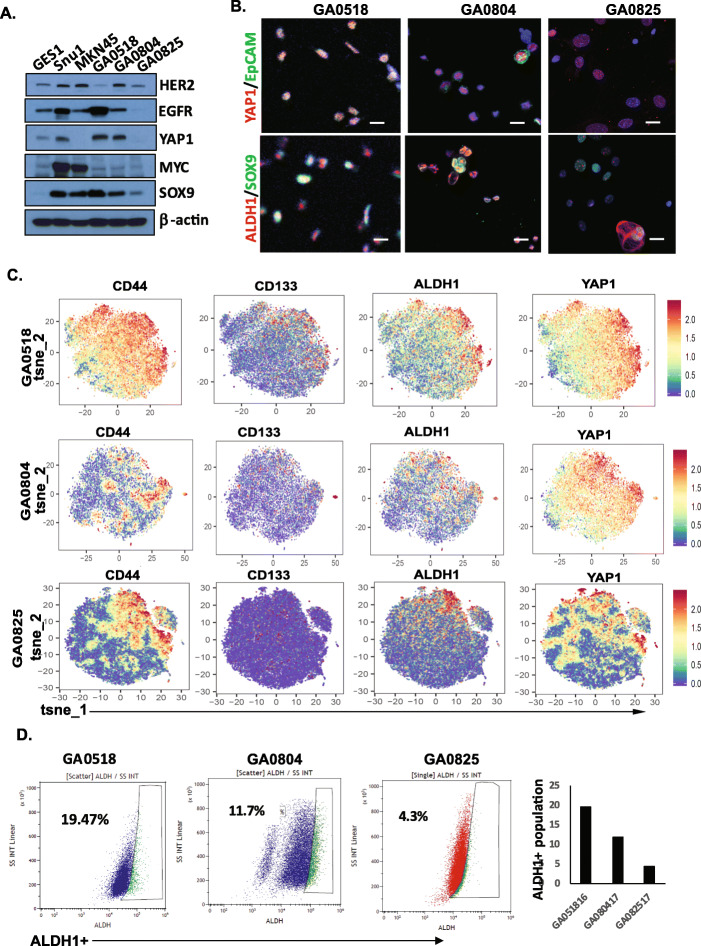
Expression of oncogenic and CSC markers in cell lines and donor PC cell samples. **A**. Expression of several notable oncogenes*—EGFR*, *HER2, YAP1*, *MYC*, and *SOX9*—was assessed by Western blotting in the three new cell lines, normal gastric epithelial cell line GES-1, and commercial GAC cell lines Snu-1 and MKN45. **B**. Expression of several notable CSC markers (YAP1, EpCAM, ALDH1, and SOX9) in the three new cell lines was determined by immunofluorescence staining as described in Materials and Methods. **C**. Expression of CD44, CD133, ALDH1 and YAP1 was evaluated by CyTOF in the three cell lines as described in Materials&Methods. **D**. ALDH1+ cells were detected using ALDEFLUOR detection kit in three cell lines. All three cell lines had varying proportions of ALDH1+ cells with the highest proportion in GA0518 compared with GA0804 and GA0825

We performed Western blot using total cell lysates from the three PC cell lines and compared the results with those of the normal gastric epithelial cell line GES-1 and the commercial GAC cell lines, Snu-1 and MKN45. GA0518 and GA0804 cells had similar expression patterns for EGFR, YAP1, MYC, and SOX9, but with higher expression of these markers in GA0518 cells (Fig. 2A). However, the expression of HER2 was higher in GA0804 and GA0825 than in GA0518 (Fig. 2A).

Immunofluorescence (IF) staining of several notable CSC makers (YAP1, EpCAM, ALDH1, and SOX9) in three cell lines (Fig. 2B) and its donor PC cells (Supplemental Fig. 2) showed expression of YAP1, EpCAM, ALDH1, and SOX9 was heterogenous and varied in three PC cell lines with highest of GA0518 cells which are similar to their donor PC cells (Fig. 2B & Supplemental Fig. 2). Expression of GAC CSC markers including CD44, CD133, ALDH1, and YAP1 were also evaluated by CyTOF in three cell lines. As shown in Fig. 2C, expression of these markers was heterogeneous among these cell lines. All four markers are relatively high in GA0518 cells, while relative lower in GA0825 cells which may reflect its tumorigenicity and metastatic potential. Further, using flow cytometry, expression of CD133, CD44 and EpCAM was examined in these three cell lines (Supplemental Fig. 3) and found almost 100% of GA0518 cells were positive for CD44, while in GA0804 cells, 65.7% were positive for CD44, 98.5% were positive for EpCAM (Supplemental Fig. 3, middle). In contrast, only 6.3% of GA0825 cells were positive for CD44, 2.6% were positive population for CD133, and occasional cells expressed EpCAM (Supplemental Fig. 3, bottom). Moreover, the three cell lines had varying proportions of ALDH+ cells, with the highest labeling percentage in GA0518 cells at 19.47% compared to 10.81 and 4.74% for GA0804 and GA0825, respectively (Fig. 2D).

### Oncogenic activation and malignant behaviors of the three cell lines in vitro

**Fig. 3:**
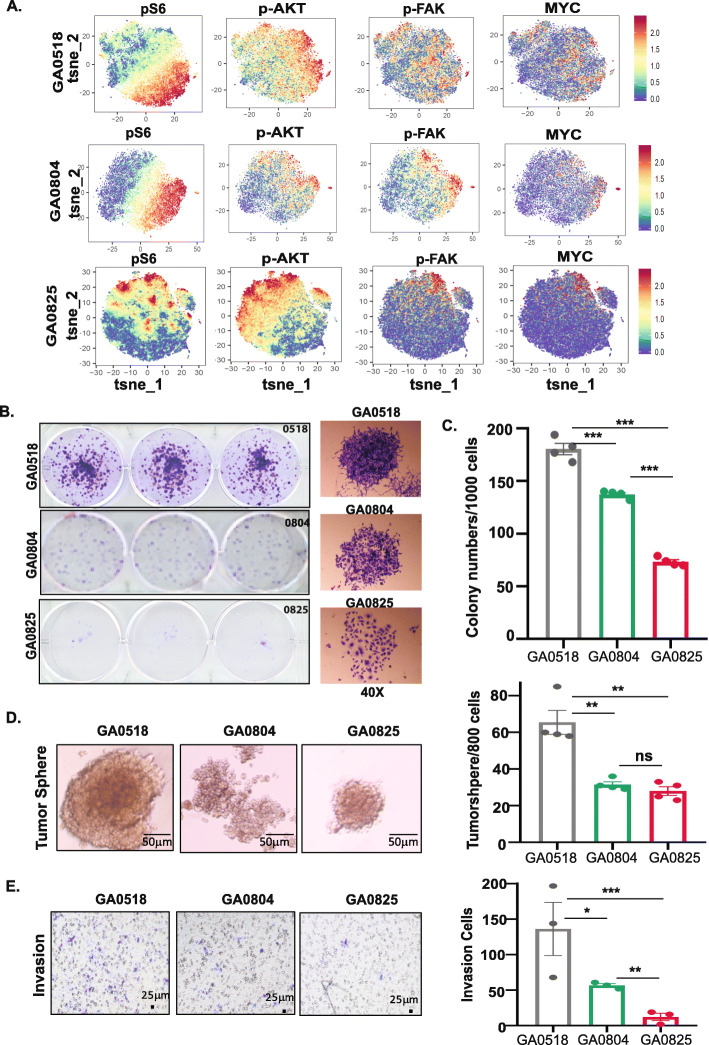
Activation of oncogenic pathways by CyTOF and malignant behaviors of three cell lines in vitro. **A**. CyTOF was performed and analyzed in the three cell lines according to the Materials and Methods. Activation of representative oncogenic pathways such as pS6, p-AKT, p-FAK and MYC was identified and varied in three cell lines. **B-E**. Malignant behaviors of three cell lines including colony formation, tumor sphere formation, and invasion were determined as described in Materials and Methods. **B** & **C**. Colony formation of three new cell lines; images of colony formation (**B**) & quantification of colony formation in three cell lines (**C**); **D**. Tumorsphere formation in three new cell lines with images (left) and quantification (right); **E**. Invasion capacity of three new cell lines with images (left) and quantification (right). Experiments were repeated at least three times. Each panel contains three triplicates. Colony number and tumor sphere number were calculated after 14 days

As the three cell lines expressed CSC markers in varying proportions, to define the similarity between the cell lines and their corresponding donor PC cells, we determined the activation oncogenic pathways in three cell lines and the corresponding donor PC cell samples by CyTOF. As shown in Fig. 3A, activation of oncogenic pathways such as pS6, p-AKT, p-FAK and mTOR and MYC were activated differently among three cell lines. GA0518 is the highest in expression of pS6, p-AKT, p-FAK and MYC which were corresponding to its donor IP-013 PC cells (Fig. 3A and Supplemental Fig. 4, top panel). The next is GA0804 and its donor PC cells IP-107-2 PC cells, while GA0825 cell and its donor IP-116 PC cells showed the lowest expression of these oncogenic markers (Fig. 3A, low panel and Supplemental Fig. 4, low panel).

We then performed functional in vitro assays in the three cell lines. The colony formation capability was highest in GA0518 (256.67 colonies/1000 cells), followed by GA0804 (105.33 colonies/1000 cells), and then GA0825 (56.33 colonies/1000 cells) (Fig. 3B & C). Both GA0518 and GA0804 formed compact colonies, while GA0825 formed diffuse colonies (Fig. 3B, left). From the images with higher magnification (40x) in Fig. 3B (right panel), we can clearly see the differences of colony patterns between GA0518 and GA0804 with packed colonies and GA0825 with loose colonies. GA0518 also had a higher tumor sphere forming capacity than GA0804 or GA0825 (Fig. 3D). GA0804 and GA0825 had similar tumor sphere forming capacity (31.5 ± 3.1 per 1000 cells for GA0804 and 28 ± 4.8 for GA0825), both of which were less than half of that of GA0518 (65.5 ± 13.0/1000 cells). While GA0518 and GA0825 formed tightly packed, round spheres, GA0804 formed loose, less rounded spheres (Fig. 3D). Both colony formation and tumor sphere formation corresponded to the ALDH1+ and CD44+ population levels with GA0518 the highest (Fig. 2). The migration capacity also varied; GA0518 had the highest capacity for invasion, followed by GA0804, and then GA0825 (Fig. 3E).

### Robust in vivo tumorigenicity in mouse xenografts

**Fig. 4:**
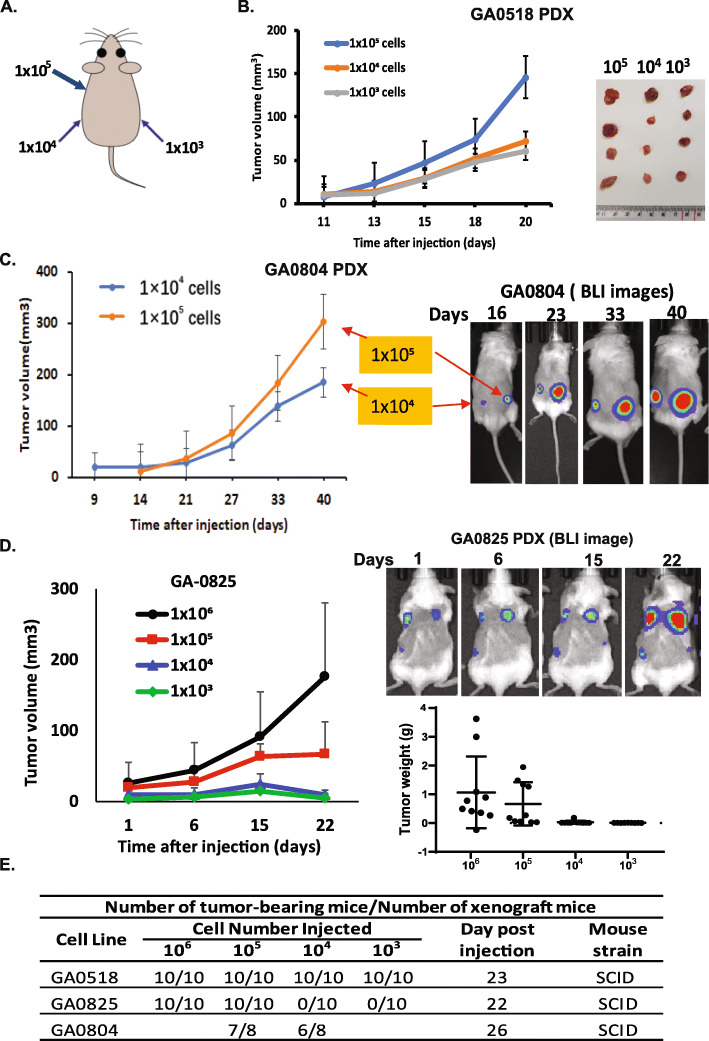
Robust in vivo tumorigenicity in patient-derived xenograft (PDX). **A**. Diagram demonstrates the varying amounts of cells injected in mice using each of the three cell lines. **B**. Representative tumor volume (left) and tumor images (right) from GA0518 cells at injected three cell doses (10^5^, 10^4^, and 10^3^ cells). **C**. Representative tumor volume (left) and tumor BLI images (right) from GA0804 cells at injected cell dosage (10^5^ and 10^4^ cells). **D**. Tumor volume (left) and representative tumor bioluminescence imaging (BLI) (right) from GA0825 cells at injected cell doses (10^6^,10^5^, 10^4^, and 10^3^ cells). Tumor growth was monitored by in vivo BLI weekly with intraperitoneal injection of 150 μg/g of D-Luciferin (PerkinElmer). Tumor weights of each group in GA0825 PDX was calculated by the end of the experiment (**D**, lower panel of right). **E**. Detailed summary of tumorigenicity of the three cell lines in mice

To test tumorigenicity, we subcutaneously injected varying numbers of limiting diluted cells from each of the three cell lines into the back flanks of either nude mice or SCID mice (Fig. 4A). All three lines formed xenograft tumors robustly, but GA0518 was more efficient than GA0804 and GA0825. GA0518 formed tumors in all nude mice injected, even with a low cell inoculation (1000 cells) (Fig. 4B), while GA0804 formed tumors in only 87.5% (7/8) of SCID mice injected with 1 × 10^5^ cells and 75% (6/8) of SCID mice injected with 1 × 10^4^ cells (Fig. 4C). GA0825 was able to produce tumors in 100% of SCID mice injected with 1 × 10^6^ and 1 × 10^5^ cells but was hard to grow when injected at 1 × 10^4^ and 1 × 10^3^ cells, although some BLI signals were visible (Fig. 4D, Supplemental Fig. 5). Furthermore, GA0518 grew faster in vivo than GA0804 and GA0825, which is consistent with the cell lines’ doubling times in vitro and their ALDH1+ populations (Figs. 1C and 2D), indicating that the GA0518 cell line was more aggressive both in vitro and in vivo (Fig. 4E).

### Molecular features and gene expression were preserved in PDXs compared to donor PC cells

**Fig. 5:**
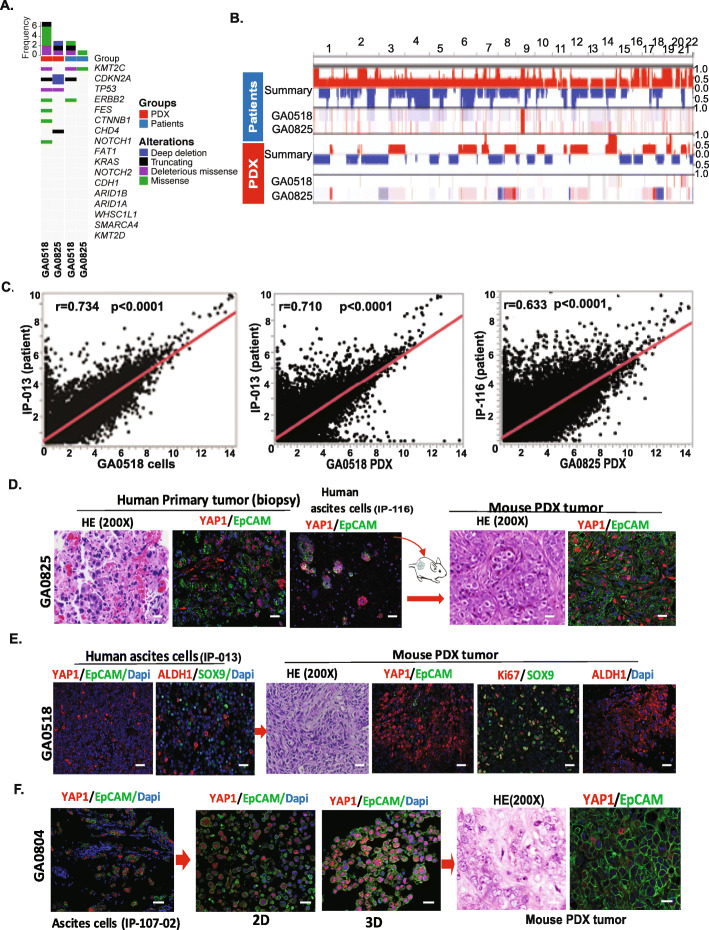
Molecular and gene expression features were preserved in PDXs compared with donor PC cells. **A** & **B**. Whole-exome sequencing was performed in GA0518- and GA0825-derived PDXs and on donor PC cells directly from patients according to the Materials and Methods. Mutations in GA0518- and GA0825-derived PDXs and donor PC cells were analyzed by a bioinformatician (S.Z.) (**A**). Copy-number variation status of GA0518- and GA0825-derived PDXs compared with the donor PC cells was analyzed as well. Common copy-number variations between PDXs and donor PC cells are shown (**B**). **C**. RNA-seq was performed in the GA0518 cell line, the corresponding donor PC cells (IP-013), and PDX tumors from the GA0518 and GA0825 cell lines, and global gene expression profiles were compared between the GA0518 cell line and donor PC cells (left), between the GA0518-derived PDX and donor PC cells (middle), and between the GA0825-derived PDX and donor PC cells (IP-116). Significantly positive correlation in global gene expression was observed for each comparison (*p* < 0.0001). **D-F**. Phenotypes of PDXs and donor PC cells were examined by hematoxylin and eosin (HE) and immunofluorescence staining for the CSC markers YAP1, EpCAM, ALDH1, and SOX9. All these markers were preserved in PDXs, recapitulating expression in the corresponding donor PC cells

We performed WES and RNA-seq of PDXs derived from GA0518 and GA0825 and of the corresponding donor PC cells directly from patients. As shown in Fig. 5A, several key mutations such as *KMT2C, CDKN2A*, and *ERBB2* mutations were preserved in the GA0518 PDXs, while GA0518 and GA0825 PDXs displayed new mutations in *TP53, FES, CTNNB1, NOTCH1*, and *CHD4*, which we have reported in the corresponding PC cells [10]. In addition, there were shared copy-number variations between the PDXs and the donor PC cells, including gains in chromosomes 1q, 7q 10p, 14q, and 19q and losses at 7p and 18q (Fig. 5B). Of note, there was a loss of *CDKN2A* in both PDXs and in the PC cells, suggesting that loss of *CDKN2A* may contribute to progression of GAC.

RNA-seq revealed a significant positive correlation in gene expression profile between the GA0518 cell line and IP-013 patient sample (*p* < 0.0001 for each (Fig. 5C, left) and between PDXs from GA0518 and the corresponding donor PC cells (IP-013) (Fig. 3C middle; p < 0.0001). Gene expression was also positively correlated between GA0825-derived PDXs and the corresponding donor PC cells, IP-116 (p < 0.0001; Fig. 5C, right), indicating that the gene signature in PDX tumors recapitulates that of the donor PC cells directly from patients.

We then compared phenotypes of PDXs and corresponding primary and PC cell samples from patients by hematoxylin/eosin staining and IF staining. As shown in Fig. 5D, for GA0825, the only cell line with a primary tumor sample available, the morphology of PDXs from mouse on hematoxylin and eosin was similar to that of the primary tumor of the patient. Interestingly, on IF staining, the expression of CSC markers YAP1 and EpCAM was preserved in GA0825 PDX, recapitulating the expression in the corresponding donor PC cells (IP-116) and its primary tumor (Fig. 5D). Similarly, expression of YAP1, EpCAM, ALDH1, and SOX9 was preserved in GA0518 PDX that recapitulate the expression in the corresponding donor IP-013 PC cells (Fig. 5E). For GA0804, the expressions of YAP1 and EpCAM was preserved in 2D and 3D culture conditions as well as GA0804 PDX from the corresponding donor PC cells (IP-107-02) (Fig. 5F). These results showed that the molecular features and gene expressions in the cell lines and PDXs had similar patterns to that of the donor PC cells from patients, suggesting that the cell lines and PDXs are potentially highly valuable to represent donor PC cells for preclinical studies.

### Improved orthotopic PDO model of PC

**Fig. 6:**
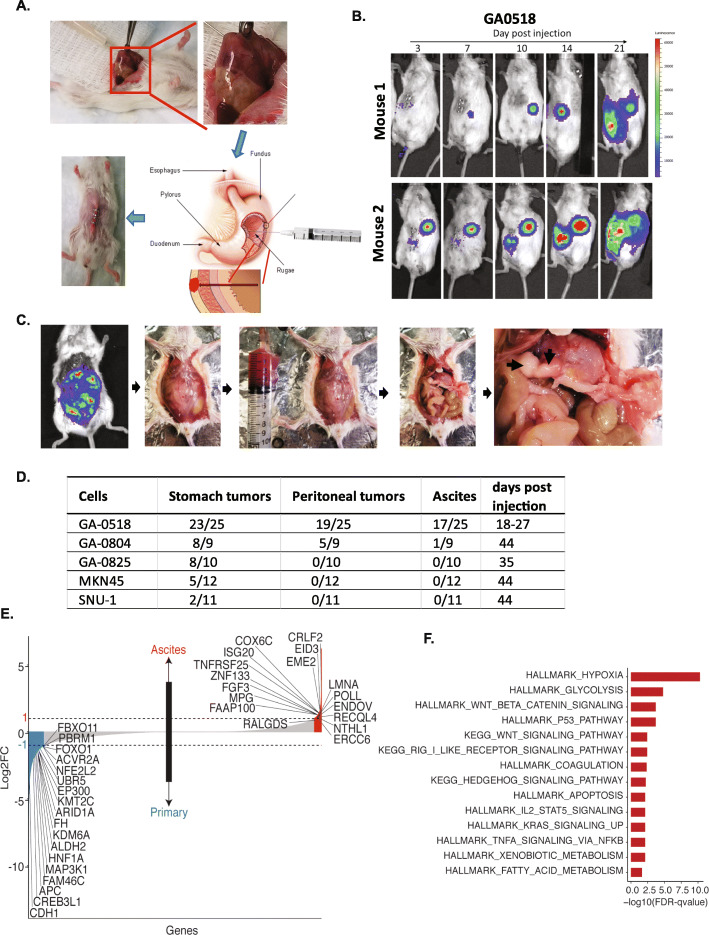
Improved orthotopic PDO model of PC. **A**. Illustration of the procedure of improved PDO model. **B**. In two representative mice, GA0518 cells generated primary stomach tumors and PC, imaged by BLI over a 3-week period. **C**. Representative PC and malignant ascites from a GA0518 cell–injected PDO model are shown. **D**. Summary of the tumorigenicity and PC metastases of the three new cell lines and two commercial GAC cell lines in our improved PDO models. **E**. RNA-seq was performed in PC cells and matching stomach tumors from the same GA0518 PDO mice as described in Materials&Methods. Significant gene changes either up or down were analyzed by bioinformatician (S.Z). F. Gene Ontology analysis was performed to identify the key genes in PC metastases involved in many important pathways, including hallmark of hypoxia, glycolysis, Wnt/β-catenin signaling, and TP53 pathways

As previously reported in GAC PDO models, GAC cell lines injected into the serosa could leak into the peritoneal cavity. To avoid this event, we changed the inoculation technique as follows: SCID mice inhaled anesthetic with isoflurane, and then the surgical site was disinfected and shaved (Fig. 6A). An upper midline incision about 1–1.5 cm long was made from the xiphoid process. The stomach was exteriorized gently using ring forceps. With the ring forceps in the left hand, the gastric antrum was lifted to reveal the posterior wall. The syringe was inserted with the right hand through the posterior wall to penetrate into the gastric cavity, and 50% Matrigel–phosphate-buffered saline containing 1 × 10^5^ cells (30 μl volume) was injected into the anterior wall, each injection lasting 30 s. After the injection, an alcohol swab was used to sterilize the posterior wall. The stomach was repositioned into the abdominal cavity, and the wound was closed (Fig. 6A).

To monitor tumor growth and metastases in vivo, we transduced all three cell lines with lentivirus expressing mCherry-Luciferase (Fig. 1B). We also used mCherry-Luciferase to label the two commercially available cell lines, Snu-1 and MKN45, which have been used for orthotopic models, for comparison [15–17]. After injection of cells, the tumor growth was monitored by BLI imaging weekly (Fig. 6B & C). In these PDO models, the three lines grew tumors in the stomach and formed PC with varying efficiency (Fig. 6B-D and Supplemental Figs. 5 and 6). GA0518 was the most aggressive, with 92% (23/25) of mice developing stomach tumors and 76% (19/25) of mice developing PC in 4 weeks (Fig. 6B-D). With GA0804, 89% (8/9) of mice developed stomach tumors and 56% (5/9) of mice had PC, while with GA0825, 80% (8/10) of mice formed stomach tumors and none (0/10) of mice had PC in about 5 weeks (Fig. 6D and Supplemental Figs. 5 and 6A). PC did not develop from commercial GAC cell lines MKN45 or Snu-1, but 42% (5/12) of mice with MKN45 and 18% (2/11) of mice with Snu-1 grew stomach tumors in 6 weeks (Fig. 6D and Supplemental Fig. 6B).

Importantly, in the PDO model, 68% (17/25) of mice with GA0518 cells developed bloody ascites within 4 weeks (Fig. 6B-D). In contrast, only 11% (1/9) of mice with GA0804 formed ascites over the course of about 6 weeks (Fig. 6D), while GA0825 and two commercial GAC cell lines MKN45 and Snu-1 did not generate PC and ascites. Further, RNA-seq of PC ascites cells and matching stomach tumors from the same GA0518 PDO mice revealed that many important oncogenes were significantly upregulated in PC cells compared with stomach tumor cells (Fig. 6E). Gene Ontology analysis identified these genes as components of many important pathways, including hallmark of hypoxia, glycolysis, Wnt/β-catenin signaling, and TP53 pathways (Fig. 6F). To understand detailed mechanisms of PC using the GA0518 PDO model warrants further investigations. These data, taken together, indicate that the GA0518 line is a good cell model to elucidate molecular mechanisms of PC and may be used for target validation.

### Sensitivity to cytotoxic agents and targeted agents in vitro and in vivo

**Fig. 7:**
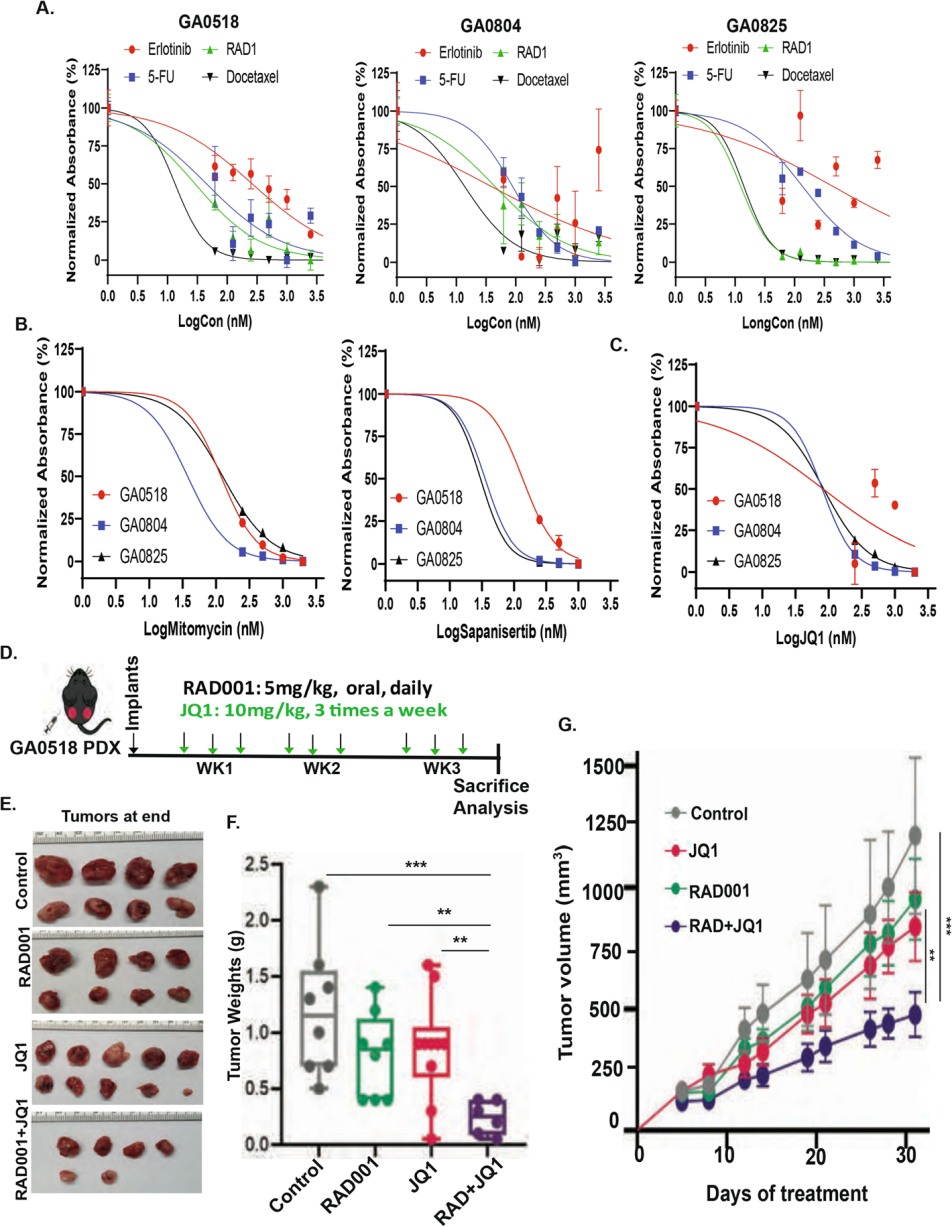
Sensitivity to cytotoxic agents and targeted agents in vitro and in vivo. **A**. MTS cell survival assays were used to examine the sensitivity of the three new cell lines to commonly used drugs including 5-fluorouracil (5-FU), docetaxel, erlotinib, and RAD001 by treating 48 h at the indicated dosage. Cell viability and IC_50_ were determined as described in Materials and Methods. **B**. The three cell lines were treated with mitomycin and a mTOR inhibitor sapanisertib, for 48 h at the indicated dosage and subjected to MTS cell survival assays. Cell viability and IC_50_ were determined as described in Materials and Methods. **C**. The three cell lines were treated with JQ1 at the indicated dosage for 48 h, and cell survival was detected using MTS assay in vitro. **D**. Diagram showed the treatment plan in GA0518 PDX in vivo. **E**. Tumor images from four group as indicated at the end of experiment. **F**. Tumor weights were shown in GA0518 PDX tumors after treated with JQ1 at 10 mg/kg alone, RAD001 3 mg/kg alone and the combination as well as control for 3 weeks. **G**. Tumor volume were calculated in GA0518 PDX.treated with JQ1 alone, RAD001 alone, or the combination for 3 weeks. **p < 0.01. ***p < 0.001

To evaluate the suitability of the cell lines and improved PDO model for drug testing, we first examined the sensitivity of the new cell lines to agents commonly used in the clinic by performing MTS cell survival assays (Fig. 7A). In addition to the cytotoxic agents 5-fluorouracil and docetaxel, we tested the targeted agents erlotinib (EGFR inhibitor) and RAD001 (an FDA improved mTOR inhibitor). As shown in Fig. 7A and Supplemental Fig. 7, the three cell lines had varied sensitivity to these four agents. GA0518 cells were highly sensitive to docetaxel, with a half maximal inhibitory concentration (IC_50_) of 13.39 nM. GA0518 cells were sensitive to RAD001 with IC_50_ of 28.91 nM but were less sensitive to 5-fluorouracil, with IC_50_ values of 45.03 nM, and were resistant to erlotinib, at an IC_50_ of 261.9 nM (Fig. 7A left and Supplemental Fig. 7A). Similarly, GA0804 cells were sensitive to docetaxel, with an IC_50_ of 13.82 nM, but less sensitive to the other three drugs (Supplemental Fig. 7B). Interestingly, GA0825 cells were highly sensitive to RAD001, with the lowest IC_50_ of 12.42 nM, and were sensitive to docetaxel, with an IC_50_ of 14.05 nM, but were resistant to the other two drugs (Fig. 7A and Supplemental Fig. 7C).

In addition, we tested several additional cytotoxic and target inhibitors, including mitomycin, sapanisertib (mTOR inhibitor), and JQ1 (inhibitor of BET family members including BRD2, BRD3, and BRD4). As shown in Fig. 7B, although responses to the inhibitors varied, the cell lines were generally the most sensitive to mitomycin and sapanisertib (mTOR inhibitor) (Fig. 7B). Cells were also relatively sensitive to JQ1 (Fig. 7C). We then validated the in vitro results in the in vivo GA0518 PDX model, studying GA0518 PDXs with JQ1 alone or in combination with RAD001, a mTOR inhibitor since we recently reported that GA0518 PDX had high mTOR activation by increased phosphor-S6 (pS6) [22]. In vivo study plan was shown in Fig. 7D with JQ1 10 mg/kg, three times a week and RAD001 5 mg/kg, oral, daily for three-week treatment (Fig. 7D). As shown in Fig. 7E-G, the combinational treatment of JQ1 and RAD001 significantly suppressed tumor weight and tumor volume and had better antitumor effects than either treatment alone. These results suggest that JQ1 in combination with RAD001 could be a potentially effective strategy worth testing in the clinic and that GA0518 cells is a good model for drug testing in vitro and in vivo.

## Discussion

We are reporting three new GAC cell lines derived from three patients with PC. All three cell lines were highly tumorigenic and aggressive in PDX and PDO models compared with commercially available GAC cell lines (Snu-1 and MKN45), and GA0518 cells were the most consistent and aggressive. WES and RNA-seq analyses revealed that the transcriptome and phenotypic features of PDXs closely recapitulated those of the donor cells from patients. Importantly, in the improved PDO model, GA0518 cells formed PC including malignant ascites in mice within 4 weeks. These findings suggest that the cell lines and PDO PC model can be reliably used for studying PC metastatic mechanisms and for target validation to advance therapeutics for GAC.

Chromosomal instability, including abnormal numbers and structural rearrangements, is a prominent feature of cancers. Through cytogenetic analysis, we found that GA0518 has a near-diploid karyotype and cytogenetic complexity. This cell line is highly heterogeneous and comprises at least four cell subtypes, including 40% of cells with a karyotype of 45,X, 20% with 46,XX,− 12,+der [21], and 10% with 46,XX,del(8p),− 21,+der [21] (Fig. 1D, left, and Supplemental Fig. 1A). The genomic heterogeneity of GA0518 cells resembles that found in donor PC cells (IP-013). In contrast to GA0518, GA0804 is highly hyper diploid and relatively homogeneous, as shown with 54 chromosomes and only one karyotype identified by cytogenetic analysis. WES showed that GA0518 donor PC cells contain more mutations (including mutations in *KMT2C, CDKN2A, ABL1*, and *SC3H12A*) than GA0825 donor PC cells (with only KMT2C mutation). It has been reported that most gastric cancers in advanced stage have abnormal chromosomes [23, 24] and chromosomal instability can promote metastases and therapy resistance [25]. In line with this notion, more frequent chromosomal aberrations such as 70% abnormal chromosomes as shown in Fig. 1D and Fig. S1B and mutations as shown in Fig. 5A and Fig. S1A in GA0518 cells accounted for its aggressive phenotype, including a short doubling time (Fig. 1C), high tumorigenicity in the PDX model (Fig. 4B & E), and high capacity to form PC (Fig. 6B-D).

Similarly, the three cell lines established from PC patients with GAC also had high heterogeneity, with multiple subclones, and one of them had high hyper-diploidy with 91–106 chromosomes [26]. Another GAC cell line from PC, gc-006-03, contained a few subclones too, with 58.5% of cells showing a modal chromosome number of 51 ± 2 [27]. From cytogenetic data of commercially available cell lines, Snu-1 and NCI-N87 had near-diploid and Snu-16 had near-tetraploid [28]. In addition, the Snu-1, Snu-16, and NCI-N87 commercial cell lines had double minutes in some cells, which was consistent with what we observed in our GA0518 and GA0804 lines.

CD44 is a well-recognized CSC marker in GAC [29–31] and is involved in distant metastases [15]. In addition, CD44 and ALDH1 are up-regulated in PC compared with primary GAC [32]. Consistent with these previous reports, CD44 and ALDH1 were detected in the three cell lines from PC. The majority of both GA0518 cells (99.9%) and GA0804 cells (75.9%) were CD44+, while all three lines were ALDH1+ on fluorescence-activated cell sorting analyses (Fig. 3), with the highest proportion of ALDH1+ cells in GA0518, which accounts for its high tumorigenicity and metastatic capacity in vivo compared with the other cell lines. In accordance with their high expression of the CSC markers, all three lines had self-renewal capability, as demonstrated by colony formation and tumor sphere formation in vitro (Fig. 3B-D) as well as tumorigenicity in vivo (Fig. 4), with GA0518 as the most aggressive line. To our surprise, even an inoculum of 1000 GA0518 cells was able to induce tumor formation consistently in SCID mice (10/10; Fig. 4E), indicating the high tumorigenicity of this cell line.

We also improved the surgical technique for the PDO model to avoid PC due to cell leakage in the peritoneal cavity. It has been well documented that the PDX and PDO models from the same source may respond differently to the same treatments [33]. Furthermore, while the PDO models of GAC using commercial cell lines or patient-derived cells are well described, PC development in mice is rare [15–17, 20, 34, 35]. As an exception, Yanagihara et al. established two lines from PC (i.e., HSC-44PE and HSC-58) and then derived five sub-lines by repeated selection. The five sub-lines had a similar frequency of metastases to that of the donor lines. However, it took ~ 100 days for these lines to produce PC, and the frequency of PC was very low, varying from 10% (1/10) to 30% (3/10) [20]. In contrast, our GA0518 line offers a distinct advantage by causing PC frequently and much faster, with 76% of mice showing PC in 4 weeks.

## Conclusions

In summary, three new GAC cell lines were established from GAC patients with PC from the United States. All three cell lines formed PDXs with high efficiency, and the molecular features of the PDXs mimicked the donor PC cells from patients, making these cell lines highly suitable for validation of targets to be advanced in human trials. Furthermore, GA0518 cells stand out in their performance as a PC metastatic model in our improved PDO model and may provide an advantage for studying metastatic mechanisms, and for target drug screening and validation by interrogating GAC for translational studies.

## Supplementary Information

**Additional file 1.** Supplemental Materias & Methods.

**Additional file 2: Supplemental Fig. 1**. Genetic alteration of three donor PC cells and cell lines. A. Whole-exome sequencing was performed in three donor PC cells IP-013 (GA0518), IP-107-02 (GA0804) and IP-116 (GA0825) directly from patients according to the Materials and Methods. Mutations in three donor PC cells were analyzed by a bioinformatician (S.Z.). B&C. Karyotyping analyses of GA0518 (B) and Snu-1 (C) were performed by the Cytogenetics and Cell Authentication Core at MD Anderson as described in Materials and Methods. Abnormal chromosomal changes were observed in GA0518 (B) and Snu-1 (C). Red arrows indicate some of the chromosomal changes in each cell lines. **Supplemental Fig. 2**. Expression of CSC markers in the corresponding donor PC cell samples. Expression of several notable CSC markers (YAP1, EpCAM, ALDH1, and SOX9) in the corresponding donor PC cell samples (IP-013, IP-107-2, and IP-116) was determined by immunofluorescence staining as described in Materials and Methods. **Supplemental Fig. 3**. Expression of CD44, CD133, and EpCAM was evaluated by flow cytometry in the three new cell lines. **Supplemental Fig. 4**. Activation of stemness and oncogenic markers in three corresponding donor PC cells by CyTOF. CyTOF was performed in the donor PC cell samples IP-013, IP-107-2, and IP-116 according to the Materials and Methods. Activation of CSC markers (CD44 and ALDH1) and oncogenic pathways such as pS6, p-AKT, and mTOR was analyzed and identified by bioinformatician (R.W). **Supplemental Fig. 5**. Representative BLI images for GA0804 PDO model A. Representative BLI images from Day 1 to Day 36 in a GA0804 PDO mouse without PC metastasis; B. Representative BLI images in GA0804 PDO mouse with PC metastasis from Day 1 to Day 36. **Supplemental Fig. 6**. Representative BLI images for GA0825 and MKN45 PDO models. A. Representative BLI images from Day 1 to Day 27 in two PDO mouse models of GA0825 with no PC. B. Representative BLI images from Day 9 to Day 26 in two PDO mouse models of commercial GAC cell line MKN45 with no PC. **Supplemental Fig. 7**. IC50 of three new cell lines on chemo and target inhibitors. The cell line GA0518 (A), GA0804 (B), and GA0825 (C) were seeded onto 96-well plates with 3000 cells/well. After overnight incubation, cells were treated with the indicated doses of two chemo-5-FU and Docetaxel; and two target inhibitors- Erlotinib and RAD001 for 6 days and then CellTiter96 Aqueous one solution was added to each well (20ul to 100ul culture media) followed by 2 h incubation at 37 °C and absorbance reading at OD 490. GraphPad Prism7 was used for IC50 analysis by following the instruction from GraphPad.com (https://www.graphpad.com/support/faq/how-to-determine-an-icsub50sub/).

**Additional file 3: Supplemental Table 1**. Patients’ characteristics.

## Data Availability

The datasets used and/or analyzed, and materials used during the current study are available to the scientific community upon request.

## Abbreviations

GAC: Gastric adenocarcinoma
PC: Peritoneal carcinomatosis
PDX: Patient-derived xenograft
PDO: Patient-derived orthotopic model
CCLE: The cancer cell line encyclopedia
WES: Whole exome sequencing
RNA-seq: RNA sequencing
CNVs: Copy-number variations
SCID: Severe combined immunodeficiency
BLI: in vivo bioluminescence imaging
CSCs: Cancer stem cells
CyTOF: Mass cytometry
TCGA: The Cancer Genome Atlas
qPCR: Quantitative real-time PCR
YAP1: Yes-associated protein 1
IC_50_: Half maximal inhibitory concentration
