# Supplementary material for: Patient-derived cell lines and orthotopic mouse model of peritoneal carcinomatosis recapitulate molecular and phenotypic features of human gastric adenocarcinoma
Source: J Exp Clin Cancer Res. 2021 Jun 23;40:207. doi: 10.1186/s13046-021-02003-8 (PMC8223395; doi:10.1186/s13046-021-02003-8)
Supplement: Supplementary file 1 — Additional file 1. Supplemental Materias & Methods. [file 13046_2021_2003_MOESM1_ESM.docx]

**Supplemental Materias&Methods:**

**Cell lines, antibodies and Reagents**

The human GAC cell lines MKN45 and Snu-1were purchased from American Type Culture Collection(ATCC, Manassas, Virginia, United States) and described previously1,2. GES-1 cells, an immortalized human gastric mucosal cell line, were described previously3 . These cells grew in RPMI-1640 medium with 10% fetal bovine serum (FBS) at 37°C in 5% CO2. All human cell lines were authenticated in the Cytogenetics and Cell Authentication Core in the Department of Genetics at The University of Texas MD Anderson Cancer Center every 6 months. The antibodies against human EGFR (Cat#4267), βeta-actin (Cat# 3700), and YAP1 (Cat# 4912) were from Cell Signaling Technology (**Beverly, MA)**; Antibody against HER2 (Cat# A0485) was from Dako (Santa Clara, CA) and antibody against SOX9 (Cat# AB5535) from EMD Millipore **(California, CA).** Antibody against c-Myc (Cat #710007) was from Invitrogen. Fluorescence-conjugated antibodies against CD44, CD133 and EpCAM for flow cytometry were from **EMD Millipore (**Billerica, MA**).** ALDH1 labeling kit ALDEFLUOR™ was from STEMCELL Technologies.

**Cell growth and doubling time**

All three established cell lines grew well in RPMI-1640 medium containing 7% FBS and 1% antibiotics (antibiotic-antimycotic, 100x). To determine the doubling time of each cell line, 1×105cells were seeded onto each well of the 6-well plates on day 0 and then the number of cells in each well was counted daily during days 1 through 4. Triplicated wells were used for each time point. The doubling times were calculated at the log phase of cell growth using the online software from <https://doubling-time.com/compute.php>. Cell morphology was analyzed by an inverted Olympus phase-contrast microscope.

**Cell survival MTT assay**

Cell proliferation was measured using CellTiter_96®_AQueous One Solution kit (Promega) following the manufacturer’s instructions. Briefly, 1000 cells in 100 µl of culture media were seeded onto each well of the 96-well plate and drugs were added the next day at various doses. Cells were incubated at 37 °C, 5% CO2 for 6 days. At the end of incubation, the medium was removed, and 120 µl of media containing 20 µl of CellTiter 96® AQueous One Solution Reagent was added to each well, followed by a 2-hour incubation at 37 °C and then absorbance reading at 490nm.

**Karyotyping analysis**

Karyotyping analyses of GA0518 and GA0804 and Snu1 cell lines were performed by the Cytogenetics and Cell Authentication Core at MD Anderson Cancer Center. Briefly, exponentially growing cells in 10 cm petri dishes were treated with colcemid (0.04μg/ml media) and incubated at 37 °C for 2 hours. Cells were then trypsinized and the single-cell suspension was collected in 15 ml conical centrifuge tubes. The cell suspension was centrifuged at 1500 rpm for 7 min. The supernatant was discarded and the cell pellet was resuspended in 8 ml of hypotonic solution (0.075 M KCl) for 20 min at room temperature. After incubation in hypotonic, 2 ml of fixative (methanol and acetic acid, 3:1 v/v) was added to the cells, which were then mixed and centrifuged. The cells were washed three times with a fresh fixative. A few drops of cell suspension were dropped onto wet glass slides and allowed to air dry. Slides were optimally aged (3 days at 65°C) and then Giemsa banded, using trypsin EDTA solution following routine laboratory techniques. G-banded metaphase spreads were photographed using an 80i Nikon Microscope and Applied Spectral Imaging (ASI) Karyotyping system. A minimum of ten metaphases were karyotyped.

**Western Blot and Flow Cytometry**

Cells grew at 75~85% confluence were trypsinized and washed once with PBS. Cell pellets were lysed with the RIPA buffer (Thermo Fisher Scientific) containing protease inhibitors (complete protease inhibitor cocktail, Roche). Equal amount of protein (i.e., about 25-30 µg) was loaded to each well for Western Blot to detect the expression levels of proteins of interest. Cell surface markers for cancer stem cells (CSCs)including CD44, CD133, and EpCAM, were determined by flow cytometry using conjugated primary antibodies against CD44, CD133, and EpCAM, respectively, while the conjugated isotype antibody was used as a control. To identify ALDH1positive cells, we analyzed the cells with high ALDH1 activity on flow cytometry using an ALDEFLUOR kit (STEMCELL Technologies).

**Colony formation assay and tumor sphere formation**

The colony formation of the new cell lines was determined using a previously reported protocol4. Briefly, 1000 cells were seeded onto the 6-well plates (in triplicate for each cell line) and incubated in normal culture conditions for 10~12 days before fixing and staining. Cell colonies from each well were counted and a bar graph with average colony number for each cell line was drawn using the Excel software.

The tumor sphere formation assay was performed as previously reported5 . In brief, 800 cells from each line were seeded onto each well of a 12-well ultralow attachment plate (in triplicate for each line) and grown in serum-free tumor-sphere medium for about 12 days. The number of tumor spheres with diameter > 100µm were counted under an inverted microscope.

**Transwell invasion assay**

The invasion assay was performed as described previously6 . Briefly, 1×105 cells in 60 µl of culture medium containing 0.5% FBS were plated on the well of transwell insert (Corning) and then 600 µl of culture medium with 20% FBS was gently added to the lower well followed by incubation of the cells at 37 °C, 5% CO2 for 48 hours. The culture media and cells inside of the insert were removed with cotton-tipped applicators and the migrated cells were fixed with 4% formaldehyde and stained with 0.2% crystal violet. The migrated cells were counted under an invert microscope.

**CyTOF Mass Cytometry**

CyTOF for three donor PC cells (IP-013, IP-107-2 and IP-116) were performed in the cellular and molecular cores as described previously7. CyTOF data were pre‐gated to exclude dead cells and irrelevant populations and concatenated using FlowJo v.10 software. t-Distributed Stochastic Neighbor Embedding (t-SNE) dimension reduction and Pheno-Graph clustering analyses were performed using open‐source R package, CyTOF kit (github.com/JinmiaoChenLab/cytofkit).

**Whole-exome sequencing analysis (WES and CNV)**

gDNAs Extracted from PC cells and the corresponding PDXs were submitted to the Sequencing and Microarray Core Facility (SMF) at UT MD Anderson Cancer Center for WES. Illumina compatible exome libraries were prepared from 200-500ng of Biorupter Ultrasonicator (Diagenode) sheared RNase-treated gDNA using the Agilent SureSelectXT Reagent Kit. MuTect (v1.1.4)8 was applied to identify somatic point mutations as previously described9.

**RNA Seq analysis**

Total RNA was isolated from PC cells and the corresponding PDXs using an miRNeasy Mini Kit (Qiagen) according to the manufacturer’s protocol from PC cells and the corresponding PDXs. Only RNA with more than 7 RNA integrity number was sent to the core for RNA Sequencing. The raw RNA-sequencing (RNA-seq) readouts were mapped to the human GRCh38 assembly reference genome using TopHat2, an open-source software tool that aligns RNA-seq reads to a reference genome. The heatmaps were generated with unsupervised clustering and the differential gene expression analyses were performed with DESeq2 (R/Bioconductor package) using adjusted p value < 0.05 as the significance cutoff.

**PDX model to study tumorigenicity**

To test tumorigenicity of the cell lines, different numbers of cells (1×103 to 1×106) from each line were subcutaneously injected into the back flanks of either nude mice or SCID mice (4-6 weeks old), and tumor growth was monitored by measuring tumor size with a digital caliper weekly. Tumor volume was calculated as (width2 × length)/2.

**References**

1. Song S, Wang Z, Li Y, et al: PPARdelta Interacts with the Hippo Coactivator YAP1 to Promote SOX9 Expression and Gastric Cancer Progression. Mol Cancer Res 18:390-402, 2020

2. Oda T, Kanai Y, Oyama T, et al: E-cadherin gene mutations in human gastric carcinoma cell lines. Proc Natl Acad Sci U S A 91:1858-62, 1994

3. Fan HN, Zhu MY, Peng SQ, et al: Dihydroartemisinin inhibits the growth and invasion of gastric cancer cells by regulating cyclin D1-CDK4-Rb signaling. Pathol Res Pract 216:152795, 2020

4. Crowley LC, Christensen ME, Waterhouse NJ: Measuring Survival of Adherent Cells with the Colony-Forming Assay. Cold Spring Harb Protoc 2016, 2016

5. Song S, Li Y, Xu Y, et al: Targeting Hippo coactivator YAP1 through BET bromodomain inhibition in esophageal adenocarcinoma. Mol Oncol, 2020

6. Justus CR, Leffler N, Ruiz-Echevarria M, et al: In vitro cell migration and invasion assays. J Vis Exp, 2014

7. Ajani JA, Xu Y, Huo L, et al: YAP1 mediates gastric adenocarcinoma peritoneal metastases that are attenuated by YAP1 inhibition. Gut, 2020

8. Cibulskis K, Lawrence MS, Carter SL, et al: Sensitive detection of somatic point mutations in impure and heterogeneous cancer samples. Nat Biotechnol 31:213-9, 2013

9. Wang R, Song S, Harada K, et al: Multiplex profiling of peritoneal metastases from gastric adenocarcinoma identified novel targets and molecular subtypes that predict treatment response. Gut 69:18-31, 2020
