# Supplementary material for: Patient-derived cell lines and orthotopic mouse model of peritoneal carcinomatosis recapitulate molecular and phenotypic features of human gastric adenocarcinoma
Source: J Exp Clin Cancer Res. 2021 Jun 23;40:207. doi: 10.1186/s13046-021-02003-8 (PMC8223395; doi:10.1186/s13046-021-02003-8)
Supplement: Supplementary file 2 — Additional file 2: Supplemental Fig. 1. Genetic alteration of three donor PC cells and cell lines. A. Whole-exome sequencing was performed in three donor PC cells IP-013 (GA0518), IP-107-02 (GA0804) and IP-116 (GA0825) directly from patients according to the Materials and Methods. Mutations in three donor PC cells were analyzed by a bioinformatician (S.Z.). B&C. Karyotyping analyses of GA0518 (B) and Snu-1 (C) were performed by the Cytogenetics and Cell Authentication Core at MD Anderson as described in Materials and Methods. Abnormal chromosomal changes were observed in GA0518 (B) and Snu-1 (C). Red arrows indicate some of the chromosomal changes in each cell lines. Supplemental Fig. 2. Expression of CSC markers in the corresponding donor PC cell samples. Expression of several notable CSC markers (YAP1, EpCAM, ALDH1, and SOX9) in the corresponding donor PC cell samples (IP-013, IP-107-2, and IP-116) was determined by immunofluorescence staining as described in Materials and Methods. Supplemental Fig. 3. Expression of CD44, CD133, and EpCAM was evaluated by flow cytometry in the three new cell lines. Supplemental Fig. 4. Activation of stemness and oncogenic markers in three corresponding donor PC cells by CyTOF. CyTOF was performed in the donor PC cell samples IP-013, IP-107-2, and IP-116 according to the Materials and Methods. Activation of CSC markers (CD44 and ALDH1) and oncogenic pathways such as pS6, p-AKT, and mTOR was analyzed and identified by bioinformatician (R.W). Supplemental Fig. 5. Representative BLI images for GA0804 PDO model A. Representative BLI images from Day 1 to Day 36 in a GA0804 PDO mouse without PC metastasis; B. Representative BLI images in GA0804 PDO mouse with PC metastasis from Day 1 to Day 36. Supplemental Fig. 6. Representative BLI images for GA0825 and MKN45 PDO models. A. Representative BLI images from Day 1 to Day 27 in two PDO mouse models of GA0825 with no PC. B. Representative BLI images from Day 9 to Day 26 in two PDO mouse mode [file 13046_2021_2003_MOESM2_ESM.pdf]

## Supplemental Figure 1.

### A.

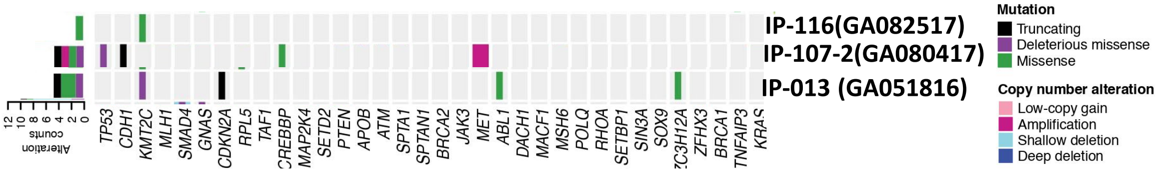


### B. Karyotyping of GA0518 cells

**45, X representative Karyotype**

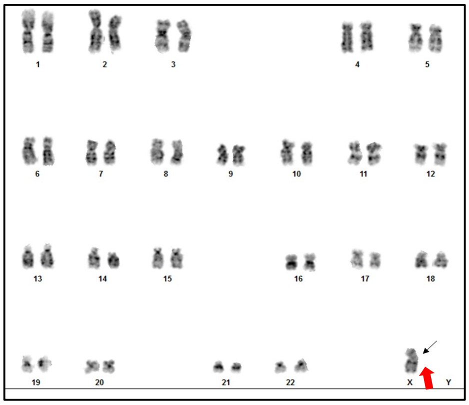


**46, XX, del(8p), -21, +der(21)**

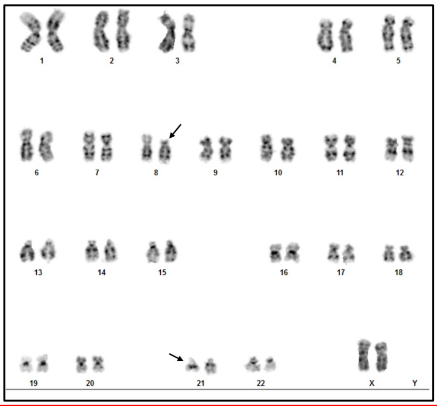


GA0518 cells are heterogeneous and about 70% cells are abnormal, including 40% cells showed 45, X karyotype, 20% cells showed 46, XX, -12, +der(21), and 10% cells showed 46, XX, del(8p), -21, +der(21).

### C. Karyotyping of Snu1 cells

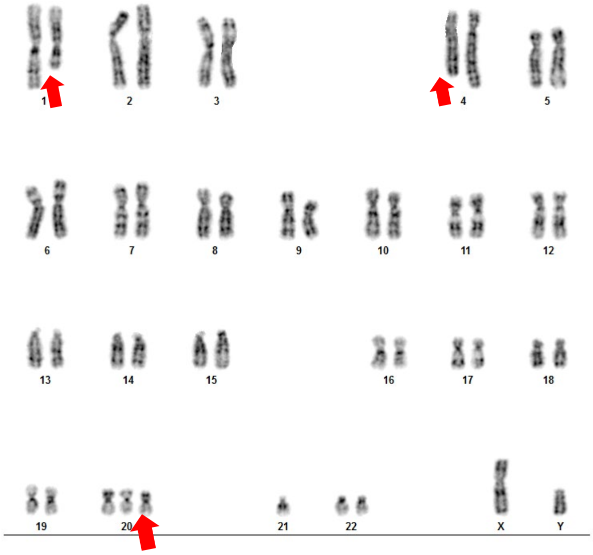

**Supplemental Figure 2.**

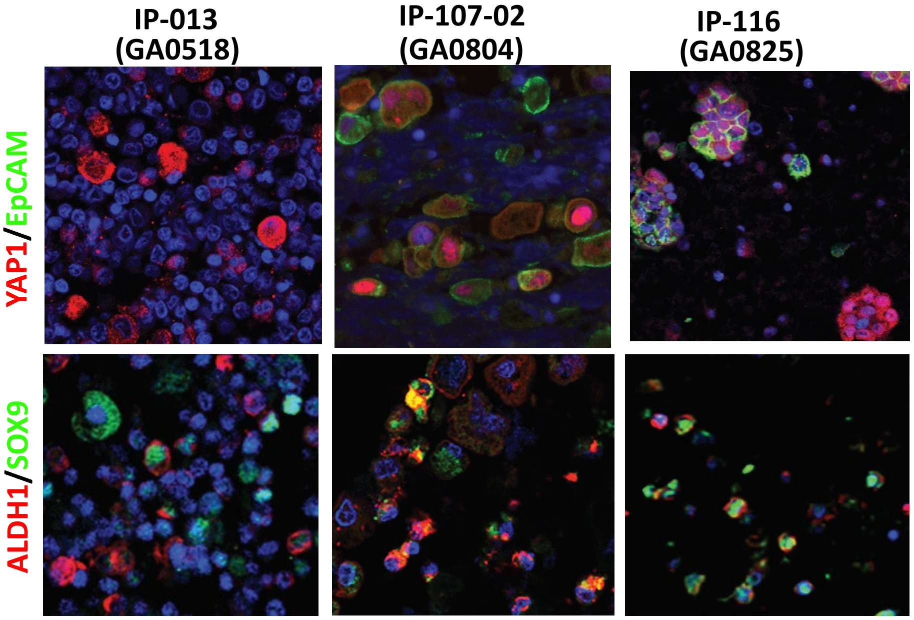

# Supplemental Figure 3.

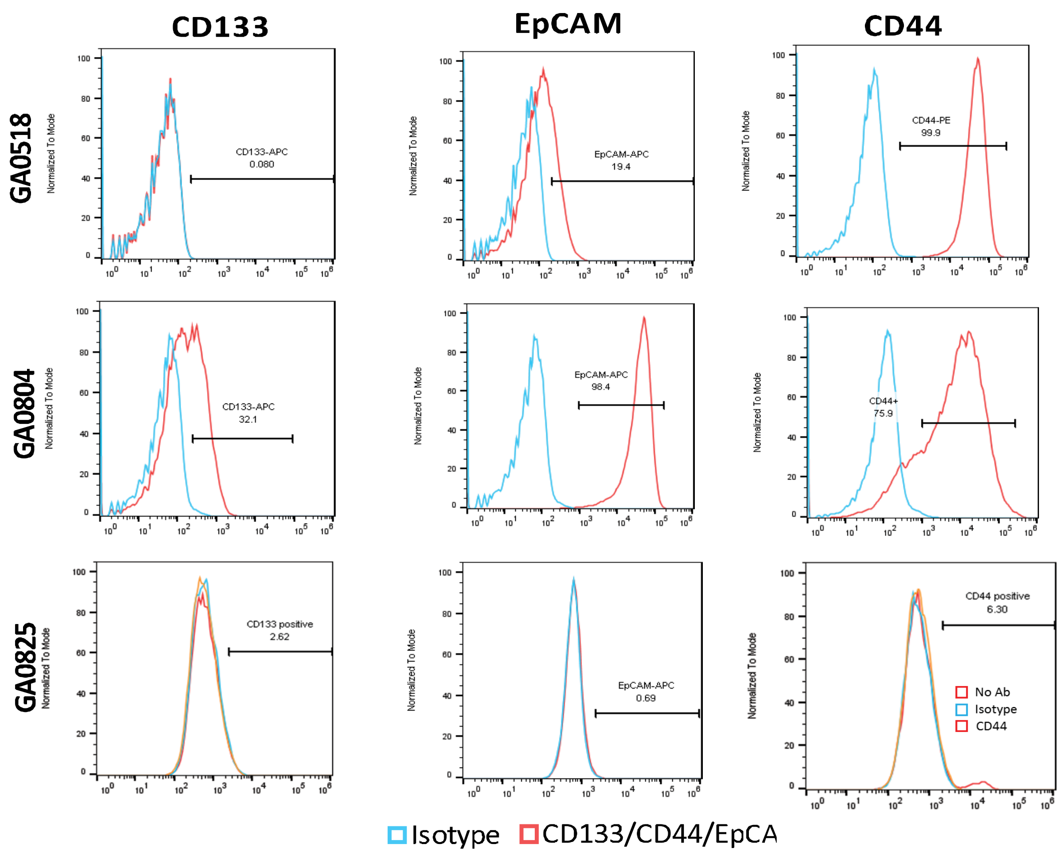

**Supplemental Figure 4.**

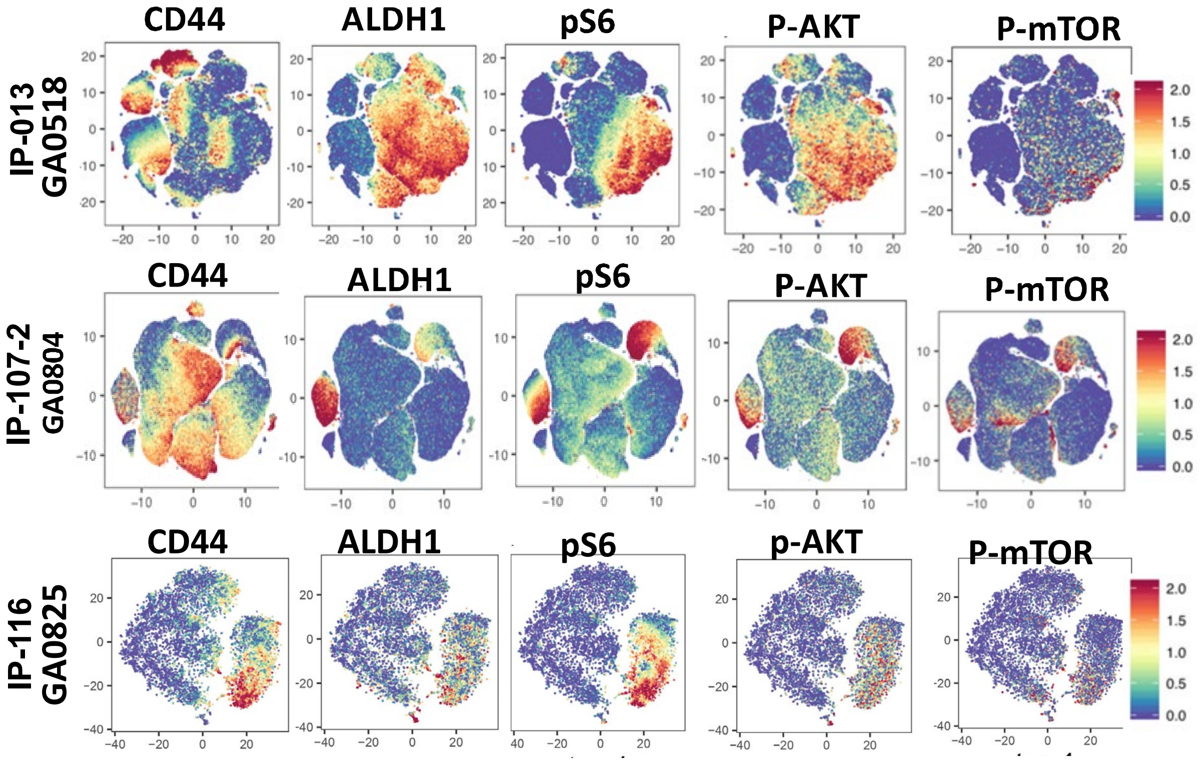

**Supplemental Figure 5.**

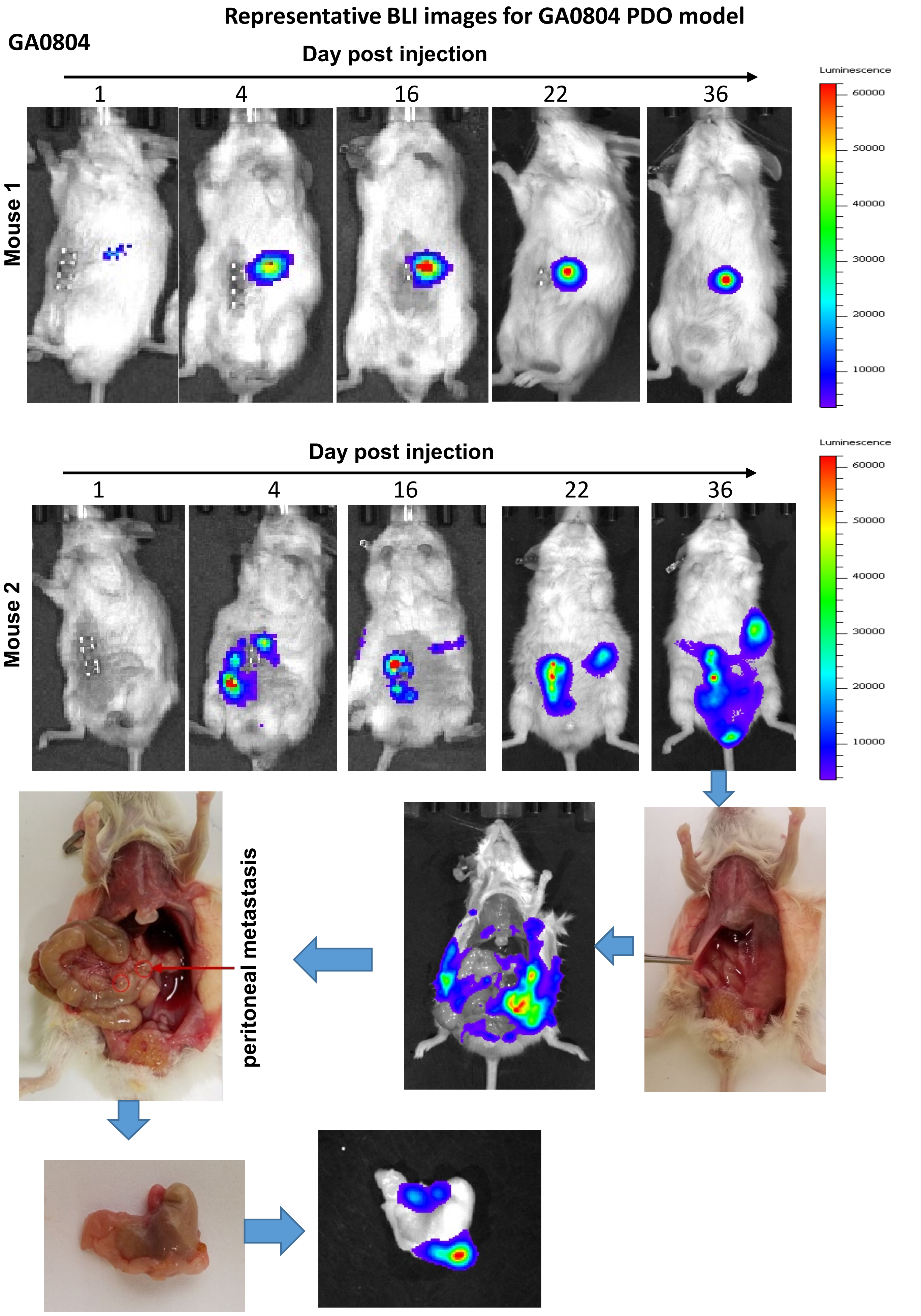

**Supplemental Figure 6.**

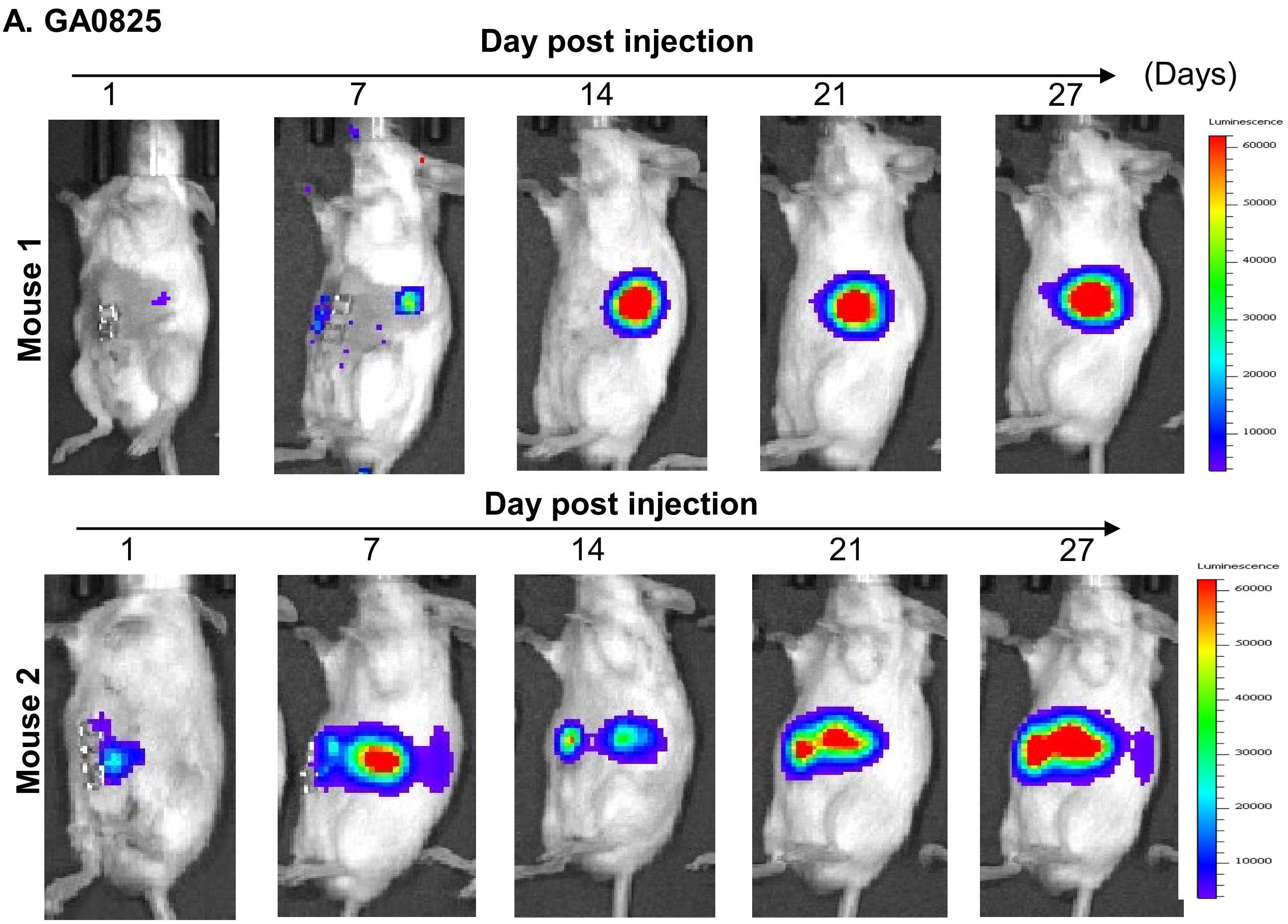


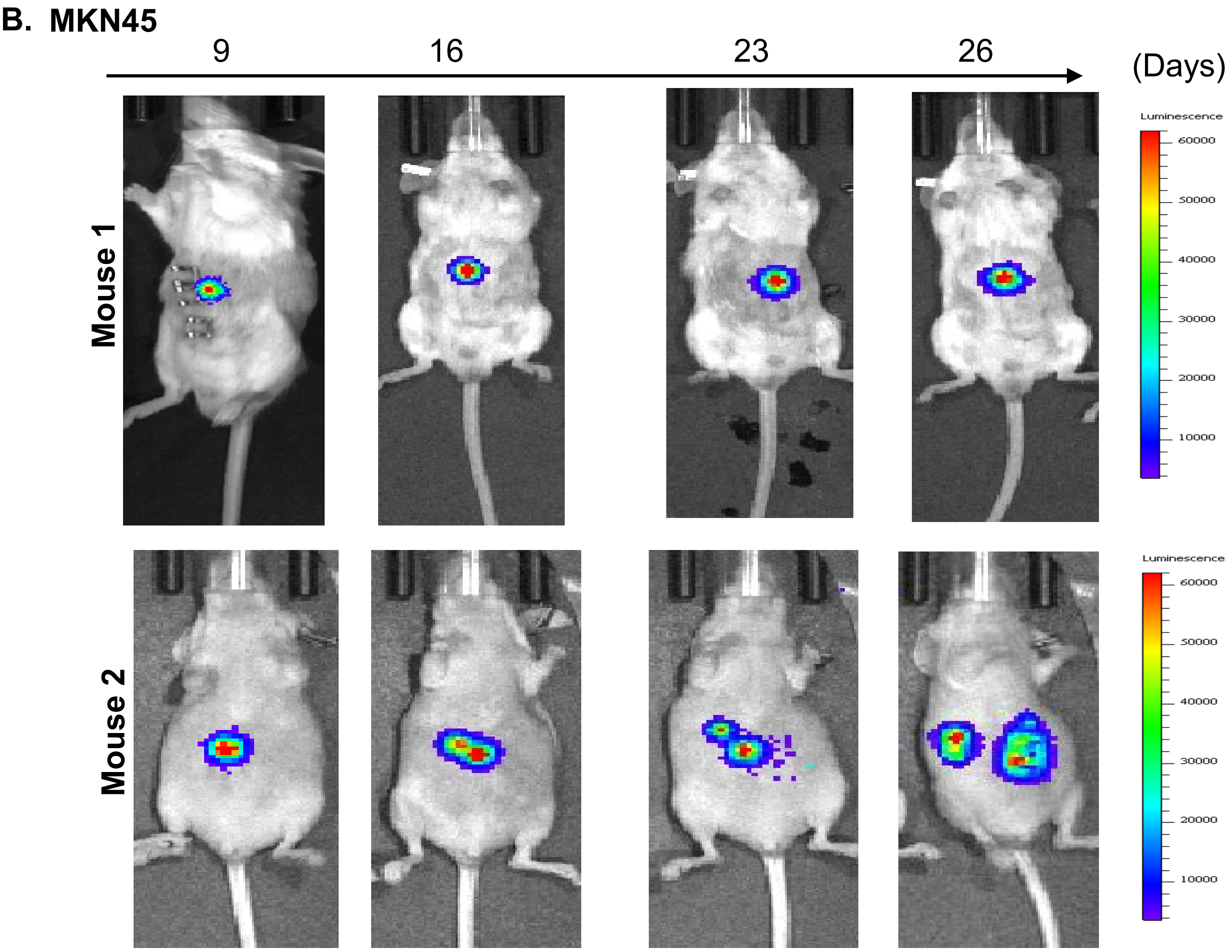

**Supplementary Figure 7.**

**A. GA0518**

IC50: 261.9nM, 45.03nM, 28.91nM, and 13.39nM for Erlotinib, 5-FU, RAD1, and Docetaxel, respectively.

$R^2$= 0.8112, 0.6646, 0.79, and 0.9894 for Erlotinib, 5-FU, RAD1, and Docetaxel, respectively.

**B. GA0804**

IC50: 38.89nM, 88.66nM, 44.76nM, and 13.82nM for Erlotinib, 5-FU, RAD1, and Docetaxel, respectively.

$R^2$= 0.2118, 0.8705, 0.7161, and 0.8024 for Erlotinib, 5-FU, RAD1, and Docetaxel, respectively.

**C. GA0825**

IC50: 439.0nM, 135.7nM, 12.42nM, and 14.05nM for Erlotinib, 5-FU, RAD1, and Docetaxel, respectively.

$R^2$= 0.3467, 0.9308, 0.9639, and 0.9909 for Erlotinib, 5-FU, RAD1, and Docetaxel, respectively.
