# Supplementary material for: Patient-derived cell lines and orthotopic mouse model of peritoneal carcinomatosis recapitulate molecular and phenotypic features of human gastric adenocarcinoma
Source: J Exp Clin Cancer Res. 2021 Jun 23;40:207. doi: 10.1186/s13046-021-02003-8 (PMC8223395; doi:10.1186/s13046-021-02003-8)
Supplement: Supplementary file 3 — Additional file 3: Supplemental Table 1. Patients’ characteristics. [file 13046_2021_2003_MOESM3_ESM.pdf]

Supplemental Table 1. Patients' characteristics

Cell lines	Ascites Information						Baseline Staging													1st-line of Treatment			
		Ascites colletion d	from ascites collection	Last follow up	Race	SEX	SITE Siewert class	Base T	Base N	Base M	location of Meta at Ascites collection	Baseline TNM	Primary Tumor Histology	Adenocarcinoma Subtype (M, SRC, NE, NOS)	Tumor Grade	Histological Type of Gastric Cancer	Her2	PD-L1	MSI	1st-line ttt	Best response to 1st-line ttt	Date of progression 1	Type of progression 1
GA0518	IP-013	5/18/2016	0.467	6/1/2016	Black	Female	Gastric	N/A	N+	M1	peritoneal, retroeritor	N/AN+M1	Adenocarcinoma	NOS	G3 Poorly diff.	NOS	N/S	N/S	N/S	No TTT			
GA0804	IP-107-2	8/4/2017	0.5	8/19/2017	Hispanic	Female	Gastric	T4	N+	M1	peritoneal, ovary	T4N+M1	Adenocarcinoma	SRC	G3 Poorly diff.	Diffuse	negative	N/S	N/S	FOX48 x4c	partial response		
GA0825	IP-116	8/25/2017	0.433	9/7/2017	White	Male	AEG I	T3	N2	M0	peritoneal, LN, Liver,	T3N2M0	Adenocarcinoma	NOS	G3 Poorly diff.	Diffuse	negative	positive	negative	Carbo/taxol +)	partial resp	11/29/2016	Liver mets
